# LGBQ+ Self-Acceptance and Its Relationship with Minority Stressors and Mental Health: A Systematic Literature Review

**DOI:** 10.1007/s10508-020-01755-2

**Published:** 2020-06-05

**Authors:** Jake Camp, Silia Vitoratou, Katharine A. Rimes

**Affiliations:** 1 Department of Psychology, Henry Wellcome Building, Institute of Psychiatry, Psychology, and Neuroscience, King’s College London, De Crespigny Park, London, SE5 8AF UK; 2 Psychometrics and Measurement Lab, Department of Biostatistics and Health Informatics, Institute of Psychiatry, Psychology, and Neuroscience, King’s College London, London, UK

**Keywords:** Self-acceptance, Minority stress, Mental health, Sexual orientation, Sexuality

## Abstract

Many individuals who identify as lesbian, gay, bisexual, queer, and with other non-heterosexual orientations (LGBQ+) experience stigma, prejudice, and/or discrimination because of their sexuality. According to minority stress and identity development theories, these experiences can contribute to difficulties with self-acceptance of sexuality. Lower self-acceptance is considered a risk factor for adverse mental health outcomes. The current review aims to investigate whether self-acceptance of sexuality is associated with minority stressors or difficulties with mental health in LGBQ+ individuals, as well as whether there are differences in self-acceptance between different sexual orientations. Five bibliographic databases were searched. Thirteen studies were identified which used quantitative methodology to investigate associations between self-acceptance, minority stressors, and/or mental health within LGBQ+ samples, or differences in self-acceptance between different sexual orientations. The results from these cross-sectional studies suggested that lower self-acceptance of sexuality was associated with higher levels of self-reported minority stressors, including a lack of acceptance from friends and family, a lack of disclosure to others, and internalized heterosexism. Lower self-acceptance of sexuality was associated with poorer mental health outcomes, including greater global distress, depression symptoms, and lower psychological well-being. There was no significant relationship with suicidality. Studies also found that LGBQ+ individuals had lower general self-acceptance compared to heterosexual participants, bisexual individuals had lower sexuality self-acceptance compared to lesbian/gay individuals, and lesbian women had lower sexuality self-acceptance compared to gay men. Given the potential importance of self-acceptance for LGBQ+ populations, further research is required with more robust methodology. Self-acceptance could be a potential target in clinical interventions for LGBQ+ individuals.

## Introduction

Many individuals who identify as lesbian, gay, bisexual, queer, and with other non-heterosexual orientations (LGBQ+) experience stressors within societal contexts which privilege heterosexuality as the normal and preferred sexual orientation (Meyer, 2003). Meyer’s minority stress theory proposes that this chronic exposure to minority stressors is responsible for the observed increased risk of mental health difficulties in sexual minority populations compared to their heterosexual peers (Ross et al., 2018; Semlyen, King, Varney, & Hagger-Johnson, 2016). Consistent with this suggestion, previous evidence suggests that increased levels of minority stressors in LGBQ+ populations are negatively associated with mental well-being (Burton, Marshal, Chisolm, Sucato, & Friedman, 2013; Gnan et al., 2019; Meyer, 2003; Pitoňák, 2017). Additionally, those with mental health difficulties are suggested to also have an increased vulnerability to the negative effects of minority stressors (Pitoňák, 2017).

Minority stress theory suggests that these minority stressors are experienced on a distal to proximal continuum (Meyer, 2003). The distal end of the continuum refers to external objective stressful events, including experiences of heterosexist prejudice, stigma, discrimination, and microaggressions. The proximal end of the continuum includes expectations of distal stressors, concealment of sexuality from others, and the internalization of negative societal attitudes. One of the proposed mechanisms for the relationship between heterosexist stigma experiences and adverse mental well-being is that the internalization of negative attitudes impairs LGBQ+ individuals’ self-acceptance of their sexual orientation (Elizur & Mintzer, 2001; Meyer, 2003). Consistent with this suggestion, cross-sectional studies with LGBQ+ individuals have found that more experiences and internalization of minority stressors are associated with lower self-acceptance of sexuality, and that lower self-acceptance is associated with greater psychological distress (Pepping, Cronin, Halford, & Lyons, 2018; Shilo, Antebi, & Mor, 2015; Woodford, Kulick, Sinco, & Hong, 2014; Yanykin & Nasledov, 2017). Correspondingly, there is some evidence that self-acceptance of sexuality may mediate the relationship between heterosexist victimization and mental health using cross-sectional data (Hershberger & D’Augelli, 1995; Woodford et al., 2014).

Self-acceptance of sexuality has been defined as accepting one’s sexuality as it is and being comfortable with this part of the self (Cass, 1979; Hershberger & D’Augelli, 1995; Perrin-Wallqvist & Lindblom, 2015). This is considered a key milestone within sexual identity development frameworks (e.g., Cass, 1979; Elizur & Mintzer, 2001). Cass’s theory suggests LGBQ+ individuals first become aware of and acknowledge their sexuality. This is the precursor to building self-tolerance and then self-acceptance of sexuality as a part of one’s identity. Self-acceptance, within this theory, is suggested to be achieved by resolving internal conflicts arising from identifying as LGBQ+ within a heterosexist society, which further allows for progression in building positive feelings and pride toward the self (identity affirmation and pride), as well as successfully integrating and valuing one’s sexuality as a part of one’s identity (identity centrality). However, this model has been criticized for suggesting a common linear progression of identity development that does not acknowledge the likely complex inter-relatedness of these processes and within group variation for people with different intersectional identities (e.g., Kaufman & Johnson, 2004).

Elizur and Mintzer’s (2001) sexual identity development theory attempts to improve on Cass’s (1979) theory by suggesting that self-acceptance is one of the three major identity tasks undertaken in concert by LGBQ+ individuals, alongside building a self-definition and coming out to others about their sexuality. Sexuality self-acceptance in this theory is suggested to be achieved through depathologizing one’s sexuality by rejecting internalized negative attitudes, improving one’s positive sense of self, increasing disclosure of sexuality to others, and developing greater participation and connectedness within the sexual minority community. This process is suggested to be further nurtured by increased access to positive self-accepting LGBQ+ peers (Elizur & Mintzer, 2001; Meyer, 2003).

Some authors consider self-acceptance of sexuality to be the inverse of internalized heterosexism, and therefore, self-acceptance is often measured using self-report instruments designed to capture internalized heterosexism or negative societal attitudes (e.g., McCarthy, Fisher, Irwin, Coleman, & Pelster, 2014; Rivers, 2004). However, identity development and minority stress theories consider self-acceptance to be a related but separate identity development process, which is negatively affected by minority stressors such as the internalization of heterosexism (Cass, 1979; Elizur & Mintzer, 2001; Meyer, 2003). While lower self-acceptance of sexuality may be a possible outcome of minority stress, greater self-acceptance has also been suggested as an important minority-specific resilience factor within qualitative research and may mitigate the deleterious effects of minority stress on mental health outcomes (Aristegui, Radusky, Zalazar, Lucas, & Sued, 2018; Bakacak & Oktem, 2014; Mimiaga et al., 2015).

Components of self-acceptance of minority sexuality, including comfort with and embracing one’s sexual identity, are positively associated with general acceptance of one’s self (Rostosky, Cardom, Hammer, & Riggle, 2018). General self-acceptance is defined as adopting a non-judgmental attitude toward the “good” and “bad” aspects of the self and is detailed in several psychological theories as a protective process for managing difficult experiences and maintaining well-being (Bernard, 2013; Ryff, 2014; Ryff, Corey, & Hughes, 2003; Williams & Lynn, 2010). Lower general self-acceptance, increased self-criticism, and more negative views toward the self are common characteristics of many mental health conditions, such as depression (Beck, Rush, Shaw, & Emery, 1987). Like self-acceptance of sexuality, accepting the entirety of one’s identity is also suggested to have an important positive effect on psychological well-being in both LGBQ+ and general populations (Bernard, 2013; Rostosky et al., 2018; Ryff, 2014; Williams & Lynn, 2010). Furthermore, in general population samples, experiences of discrimination (e.g., being treated differently to others) and victimization (e.g., being threatened or harassed) generally or due to participants’ ethnicity, weight, or appearance were also found to be associated with lower levels of general self-acceptance (Ryff, 2014; Ryff et al., 2003). It might be anticipated that minority stressors would show a stronger association with self-acceptance of sexuality than with general self-acceptance, although the authors are not aware of studies that have made this direct comparison.

Despite the proposed impact of minority stressors on general and sexuality self-acceptance, and the hypothesized importance of self-acceptance for mental well-being in LGBQ+ individuals, there has been no previous review of the research evidence regarding the association between self-acceptance, minority stressors, and mental health in this population. There has also been no review of the differences in self-acceptance between different sexual orientations. Therefore, this review aims to answer the following questions: (1) Is self-acceptance of sexuality or general self-acceptance statistically associated with minority stress and mental health or well-being in LGBQ+ individuals, and (2) Are there statistical differences in general or sexuality self-acceptance between different sexual orientations? This review will include an assessment of methodological quality to assess the included studies’ design, reporting, and attempts to reduce bias, to inform conclusions.

## Method

### Search Strategy

A systematic search of the literature (PROSPERO ref: CRD42018084387) was conducted using the PRISMA strategy (Moher et al., 2015). Searches were conducted through OvidSP and Web of Science (WoS) for the following databases: Ovid MEDLINE(R) (1946 to the date of search), Embase Classic and Embase (1947 to the date of search), PsychARTICLES, PsychINFO (1806 to the date of search), and WoS Core Collection (1900 to the date of search). The initial search took place in February 2018 and was updated in March 2020.

The search terms and Boolean operators are shown in Appendix 1. Boolean operators were adapted to their equivalent form in WoS. Proximity Boolean operators (ADJ12/NEAR12) were utilized for the general term of acceptance (“Accept*”) to reduce the number of false positive results, as after discussions between the research team it was considered that “acceptance” would likely be within a maximum of 12 words of the sexual orientation terms. This search was applied to abstracts, keywords, and titles of the articles. Hand searches were conducted on the reference sections of the full text review articles and any meta-analyses/systematic reviews found in the search. Grey literature was excluded as these have not undergone peer review and are therefore not bound by high standards of quality, which could limit the ability to draw firm conclusions (Adams, Smart, & Huff, 2017).

### Selection Criteria

Studies were included if they met the following criteria:

1. Reported quantitative data on self-acceptance of sexuality or general self-acceptance, specifically for LGBQ+ groups. Self-acceptance of sexuality was measured using instruments that included items enquiring directly about participants’ acceptance and/or comfort with their sexuality.
2. Reported the results of statistical analyses investigating the relationship between the self-acceptance measure with sexual minority stressors or a mental health outcome, or if the study reported results of analyses investigating differences in self-acceptance between different sexual orientations. Minority stress measures suitable for inclusion were distal stressors (e.g., victimization, stigma, prejudice, lack of acceptance and support by others, and microaggressions) or proximal stressors (e.g., internalized stigma, expectations of distal stressors, and concealment of sexuality; Meyer, 2015). Disclosure of sexuality to others was included as a behavioral proxy measure for concealment; it is acknowledged that these processes are not considered two ends of a single continuum, but that disclosure can reflect the absence of concealment (e.g., Schrimshaw, Siegel, Downing, & Parsons, 2013, Uysal, 2019). Mental health outcomes suitable for inclusion were quantitative measures of comprehensive symptoms or diagnoses of mental illnesses or problematic substance use, and measures of global psychological distress and well-being. No other restrictions were placed on study design.
3. Published within a peer-reviewed journal.
4. Not a systematic review or meta-analysis.
5. Available in English.

Studies were excluded if they: (1) only reported data on self-acceptance in relation to other specific contexts (e.g., body self-acceptance and mindfulness acceptance); (2) self-acceptance of sexuality was measured using instruments enquiring about other distinct processes (e.g., internalized heterosexism) and/or measures did not include items enquiring about acceptance or comfort with one’s sexuality; (3) only measured specific psychological constructs (e.g., self-esteem) rather than mental health illnesses or global distress/well-being; and (4) only had qualitative research methodology. No restrictions were placed on the age of participants.

### Selection Process

References from the initial search were exported to EndNote, and duplicates were removed. All grey literature and book chapters were removed. Remaining titles and abstracts were then screened by the first author. Full texts for potentially relevant articles were retrieved and screened against the eligibility criteria both by the first author and an independent researcher who were both blind to one another’s ratings. Where there were any discrepancies between the raters in inclusion or exclusion at this stage, the two researchers resolved these through discussion with the support of the one of the senior authors (KR).

### Data Extraction

Information extraction included: citation; study design; sample size and characteristics; measurements of self-acceptance, sexual orientation, mental health outcomes, and minority stressors; and relevant analyses and results.

### Quality Assessment

Study quality was assessed using the AXIS critical appraisal tool for cross-sectional studies (Downes, Brennan, Williams, & Dean, 2016). This consisted of 20 items assessing the quality of reporting, quality of study design, and possible risk of bias. Example items for each respective subscale were “was the target/reference population clearly defined,” “was the study design appropriate for the stated aim(s),” and “was the selection process likely to select subjects/participants that were representative of the target population…” Answers were scored as *yes* (1), *no* (0), or *unable to determine* (0) with a score range of 0–20; higher scores indicated higher methodological quality and lower risks of bias. Quality ratings were only made for the parts of the studies relevant to this review (i.e., aspects including self-acceptance, mental health outcomes, and minority stressors). Two researchers completed the AXIS tool for all studies; both blind to one another’s ratings. The raters initially agreed on 87% of the ratings. The discrepant ratings were resolved by discussing with one of the senior authors (KR); consensus ratings for methodological quality are reported in Appendix 2.

### Data Synthesis

A narrative synthesis of relevant results in the included studies was completed rather than a meta-analysis. This is due to the differences and limitations in the research and statistical methodology, as well as the small number of studies investigating the outcomes, which would significantly impact the reliability of effect and variance/error estimations (e.g., Borenstein, Hedges, Higgins, & Rothstein, 2011). The narrative synthesis was aided by using a spreadsheet containing the results from each included study and discussion between research team members.

## Results

### Study Selection

**Fig. 1:**
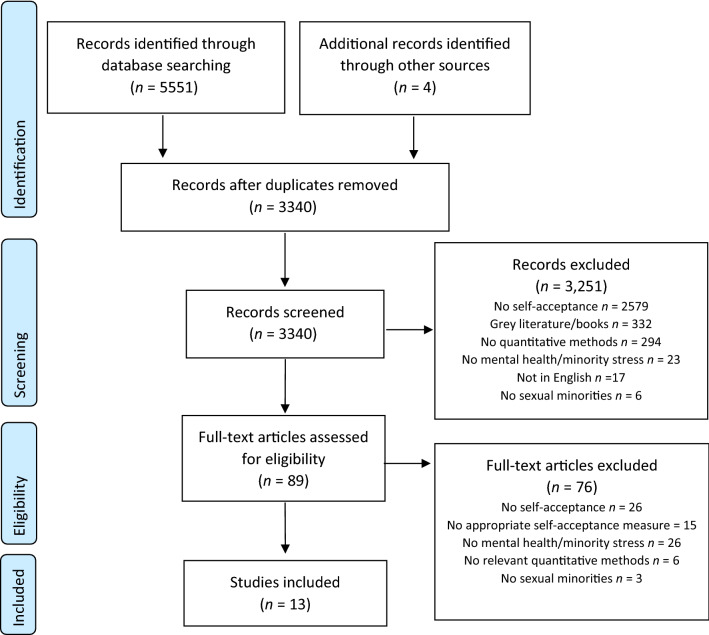
PRISMA (Moher et al., 2015) flow diagram

The search produced 3340 relevant results after removing duplicates (Fig. 1 for PRISMA flow diagram). A full text review was completed for 89 articles. Most papers were excluded at this stage because they did not include measures of self-acceptance, or there was no quantitative analysis investigating the relationship between self-acceptance and mental health outcomes or minority stressors, despite including these outcomes within separate analyses. A number of studies that suggested they investigated self-acceptance but used measures of other distinct constructs (e.g., internalized heterosexism), or that did not include items enquiring about acceptance and/or comfort with one’s sexuality, were also excluded. Eighty-five of the 89 full texts reviewed had full agreement in their inclusion/exclusion between the two raters. The inclusion/exclusion of the remaining four papers was resolved through discussion with one of the senior authors (KR). A total of 13 studies were included in this review.

### Study Characteristics

The study characteristics are summarized in Table 1. The year of publication ranged from 1979 to 2018. All included studies utilized a cross-sectional survey design and all except one used convenience or snowball sampling from sexual and gender minority community sources. The majority of studies were conducted in the U.S. or Israel. All studies used self-report measures for the variables of interest, all with varying degrees of psychometric validation. Across these studies, there were a total of 6894 participants. The average age across studies was 27.59 years (SD = 7.45; range = 12–72). Around half of the participants identified as male and the other half as female. There was insufficient information in all studies to determine whether participants’ gender identity was the same or different as their sex assigned at birth. Most participants identified as LGBQ+ (82%), of which 82% identified as lesbian or gay, 14% as bisexual, and 4% with other minority orientations (e.g., pansexual, queer). Seventy-eight percent of participants were white and the remaining 22% were from other race-/ethnicity-related groups. The aforementioned demographic summaries were calculated only for studies that presented sufficient demographic information.

**Table 1:** Characteristics of the included studies

References	Country of origin	Year data collected	Sample source	*N*	Sexual orientation	Gender	Ethnicity	Age (years) *M* (SD); range
Elizur and Mintzer (2001, 2003)	Israel	NS	Gay meeting places, clubs, associations, HIV testing clinics, snowballing	121	100% gay	100% men	89% Israeli	32
							11% other	23−72
Gil (2007)	Israel	NS	Undergraduate psychology cohort and university gay student support group	180	42% gay	100% men	80% Israeli	23 (1.6)
					58% heterosexual		20% other	
Hershberger and D’Augelli (1995)	USA	NS	Lesbian and gay community centers	165	100% lesbian, gay, or bisexual	25% women	67% White	19 (1.5)
						75% men	33% BAME	15−21
Ifrah et al. (2018)	Israel	2004	Gay youth organization	202	38% lesbian	38% women	90% Israeli	21 (4.7)
					62% gay	62% men	10% other	
Leserman et al. (1994)	USA	NS	Health departments, gay organizations, advertisements, and snowballing	169	100% gay	100% men	NS	31 (6.3) 18−50
Riggle et al. (2009)^a^	USA	1995	National	3552	3% lesbian, gay, or bisexual	50% women	87% White	44
					97% heterosexual	50% men	13% BAME	
Rosario et al. (2006)	New York, USA	1993−1995	LGBTQ + community and university organizations	156	64% consistently lesbian/gay	49% women	22% White	18 (1.6)
					20% transitioned from bisexual to gay/lesbian	51% men	78% BAME	14−21
					16% consistently bisexual			
Rosario et al. (2009)^b^	New York, USA	1993−1995	LGBTQ + community and university organizations	68	43% masculine lesbian	100% women	20% White	18 (1.6)
					27% feminine lesbian		80% BAME	14−21
					30% feminine bisexual			
Shilo and Mor (2014)	Israel	2010	General and LGBTQ + social media groups and web forums	685	21% lesbian	36% women	NS	22 (4.7)
					57% gay	64% men		
					22% bisexual			
Shilo and Savaya (2011)	Israel	2006	LGBTQ + youth groups, web forums, and snowballing	461	74% lesbian/gay	50% women	NS	18 (1.8)
					26% bisexual	50% men		16−23
Shilo et al. (2015)	Israel	2010	LGBTQ + social media groups and web forums	890	79% lesbian/gay	48% women	NS	32
					15% bisexual	52% men		12−60
					5% questioning			
					1% queer			
Siegelman (1979)	UK	NS	Newspaper advertisements, lesbian organizations, university students, and snowballing	110	63% lesbian	100% women	NS	35
					37% heterosexual			
Yanykin and Nasledov (2017)	Russia	NS	Online LGBTQ + communities and social networks.	92	100% lesbian/gay	NS	NS	29

NS = not specified, BAME =  Black and Asian Minority Ethnic groups. All included studies were cross-sectional survey designs

^a^Riggle et al. (2009) used national probability sampling, but all other studies utilized convenience and/or snowball sampling

^b^Subsample used from Rosario et al. (2006)

### Study Results

Table 2 summarizes the findings and effect sizes relating to the relationship LGBQ+ self-acceptance had with minority stressors and mental health outcomes. Table 3 summarizes the results and effect sizes from studies comparing self-acceptance between different sexual orientation-related groups. These findings are summarized and discussed in “Summary of Study Findings and Discussion” section.

**Table 2:** Relationship between sexuality self-acceptance and minority stress or mental health: Summary of measures and results

Minority stressor or mental health independent variable	Study	Independent variable measure	Self-acceptance of sexuality measure	Analysis	Relationship with self-acceptance of sexuality	Effect size
*Distal stressors*						
Lack of acceptance by family	Elizur and Mintzer (2001, 2003)	SRQ	SAQ	Correlation^a^	0	*r *= − .08
				Partial correlation (while controlling for friends’ acceptance)	0	*r *= .04
	Leserman et al. (1994)	Items developed by the authors	Modified subscale from CCM	Pearson’s correlation	−	*r* = − .19*
	Shilo and Savaya (2011)	SRQ	SAQ	Correlation^a^	−	*r* = − .24**
				Structural equation modeling	−	*β *= − .19**
	Hershberger and D’Augelli (1995)	Items developed by the authors	An item developed by the author	Correlation^a^	−	*r* = − .18*
Lack of acceptance by friends	Elizur and Mintzer (2001, 2003)	SRQ	SAQ	Correlation^a^	−	*r *= − .23*
				Partial correlation (while controlling for family acceptance)	−	*r* = − .22*
	Shilo and Savaya (2011)	SRQ	SAQ	Correlation^a^	−	*r *= − .25**
				Structural equation modeling	0	NSA
Heterosexist victimization	Hershberger and D’Augelli (1995)	VS	An item developed by the author	Correlation^a^		
				Verbal insults and/or threats	+	*r* = .17*
				Personal property damage and/or chased, followed, or spat on	+	*r* = .21*
				Physical and or sexual assault	0	*r* = .14
*Proximal stressors*						
Disclosure to others	Elizur and Mintzer (2001, 2003)	DQ	SAQ	Correlation^a^	+	*r *= .27**
				Stepwise regression (SAS and sexual orientation combined)	+	*R*^2^ = .12**
				Stepwise regression (controlling for family and friends’ support)	0	NSA
	Ifrah et al. (2018)	GIQ-SD	GIQ-AH	Correlation^a^		
				Gay men	+	*r* = .43**
				Lesbian women	+	*r *= .52**
	Shilo and Savaya (2011)	DQ	SAQ	Correlation^a^	+	*r *= .33**
Disclosure to family	Elizur and Mintzer (2001, 2003)	Items developed by authors	SAQ	Correlation^a^	+	*r* = .29**
	Leserman et al. (1994)	Items developed by the authors	Modified subscale from CCM	Pearson’s correlation	+	*r* = .25*
	Hershberger and D’Augelli (1995)	Items developed by the authors	An item developed by the author	Correlation^a^	+	*r* = .44*
Internalized heterosexism	Yanykin and Nasledov (2017)	Modified MIHI-HS	Modified MIHI-SAS	Spearman’s rho correlations	−	*ρ* = − .50**
*Mental health*						
Psychological distress	Hershberger and D’Augelli (1995)	BSI-GSI and PS	An item developed by the author	Correlation^a^		
				BSI-GSI	−	*r* = − .26*
				PS	−	*r* = − .46*
	Shilo et al. (2015)	MHI	SAQ	Multiple Regression Model 1		
				Controlling for religiosity, disclosure, victimization, family and friends’ support, LGBQ+ connectedness, and relationship status	−	*β* = − .11** *B* = − 4.09** *R*^*2*^ = .19**
				Addition of the interaction effects between the above variables and age groups (youths and adults) added to the model	0	*β* = − .08 *B* = − 3.01 *R*^*2*^ = .25**
				Multiple Regression Model 2		
				Controlling for religiosity, disclosure, and victimization		
				Young people	−	*β* = − .23** *B* = − 8.51** *R*^*2*^ = .09**
				Adults	−	*β* = − .16** *B* = − 6.29** *R*^*2*^ = .11**
				Addition of family and friends’ support, LGBQ+ connectedness, and relationship status		
				Young people	−	*β* = − .21** *B* = − 6.92** *R*^*2*^ = .22**
				Adults	−	*β* = − .09* *B* = − 3.28** *R*^*2*^ = .20**
	Shilo and Mor (2014)	MHI	SAQ	Pearson’s correlation	−	*r* = − .21**
				Multivariate Regression (controlling for age, gender, disclosure, distal and proximal harassment, family and friends’ support, and LGBQ+ connectedness)	−	*B* = − 8.04 *R*^*2*^ = .23
	Shilo and Savaya (2011)	MHI	SAQ	Correlation^a^	−	*r* = − .22**
	Yanykin and Nasledov (2017)	SCL-90-R-SMI	Modified MIHI-SAS	Spearman’s rho correlations	0	*ρ *= − .10
Psychological well-being	Shilo et al. (2015)	MHI	SAQ	Linear regression		
				Controlling for religiosity, disclosure, victimization, family and friends’ support, LGBQ+ connectedness, and relationship status		
				Young people	+	*β* = .11* *B* = 2.33** *R*^*2*^ = .28**
				Adults	+	*β* = .12** *B* = 3.13** *R*^*2*^ = .18**
				Multiple regression		
				Controlling for religiosity, disclosure, victimization, family and friends’ support, LGBQ+ connectedness, and relationship status	+	*β* = .12** *B* = 2.78** *R*^*2*^= .19** *
				Addition of the interaction effects between the above variables and age groups (youths and adults) added to the model	+	*β* = .13** *B* = 3.15** *R*^*2*^= .23*
	Shilo and Mor (2014)	MHI	SAQ	Pearson’s correlation	+	*r *= .18**
				Multivariate Regression (controlling for age, gender, disclosure, distal and proximal harassment, family and friends’ support, and LGBQ+ connectedness)	+	*B* = 3.69** *R*^*2*^ = .19**
	Shilo and Savaya (2011)	MHI	SAQ	Correlation^a^	+	*r *= .21**
	Yanykin and Nasledov (2017)	SWS	Modified MIHI-SAS	Spearman’s rho correlation	0	*ρ *= .07
Depression symptoms	Leserman et al. (1994)	CRS-D and POMS-D	Modified subscale from CCM	Stepwise regression (controlling for age, race, education)		
				HIV-Negative Gay Men		
				CRS	−	*β* = − .36** *R*^*2*^ = .16**
				POMS-D	0	NSA
				HIV-Positive Gay Men		
				CRS	0	NSA
				POMS-D	0	NSA
Suicidality	Hershberger and D’Augelli (1995)	Items developed by the authors	An item developed by the author	Correlation^a^		
				Suicidal ideation	0	*r* = − .05
				Suicide attempts	0	*r* = .10

0 = no significant association with self-acceptance; + = significant positive association with self-acceptance; − = significant negative association with self-acceptance. Statistically significant results are bolded. *p < .05; **p < .01. NSA = non-significant association, effect size not specified. *r* = correlation coefficient. *R*^2^= *r* squared. *ρ* = Spearman’s rho coefficient. *Β *= Beta. *β *=standardized Beta. *Self*-*acceptance of sexuality measures:* CCM = Modified Coping and Change Measure (Leserman et al., 1994); Gay Identity Questionnaire, Acceptance of Homosexuality Subscale (Brady & Busse, 1994); MIHI-SAS = Modified Mayfield Internalized Homonegativity Inventory, Self-Acceptance Subscale (Yanykin & Nasledov, 2017); SAQ = Self-Acceptance Questionnaire (Elizur & Mintzer, 2001). *Independent variable measures:* BSI-GSI = General Severity Index generated from the Brief Symptom Inventory (Derogatis, 1993); CRS-D = Carroll Rating Scale for Depression (Carroll, Feinberg, Smouse, Rawson, & Greden, 1981); DQ = Disclosure Questionnaire (Ravitz, 1981); GIQ-SD = Gay Identity Questionnaire, Self-Disclosure Subscale (Brady & Busse, 1994); MHI = Mental Health Inventory (Veit & Ware, 1983); MIHI-HS = Modified Mayfield Internalized Homonegativity Inventory, Homonegativity Subscale (Yanykin & Nasledov, 2017); PS = Problems Scale (Mapou, Ayres, & Cole, 1983); POMS-D = Profile of Mood States−Depression (McNair, Lorr, & Droppleman, 1981); SCL-90-R-SMI = Symptom Checklist-90-Revised, Symptomatic Manifestation Index (Derogatis, 1993); SRQ = Societal Reactions Questionnaire (Ross, 1985); SWS = Subjective Well-being Scale (Sokolova, 1996); VS = Victimization Scale Modified from Pilkington and D’Augelli (1995)

^a^Correlation type not specified

**Table 3:** Group comparison studies: Summary of measures and results

Study	Group 1	Group 2	Self-acceptance measure	Analysis	Results	Effect size
*Comparisons of general self*-*acceptance*						
Gil (2007)	Gay men	Heterosexual men	GSA: PWS-SAS	Independent samples *t* test	Gay men had lower SAS than heterosexual men	*d* = 0.44**
Riggle et al. (2009)	LGBQ+	Heterosexual	GSA: PWS-SAS	Independent samples *t* test	LGBQ+ individuals had lower SAS than heterosexual individuals	*d* = 0.20*
Siegelman (1979)	Lesbian women	Heterosexual women	GSA: DSA	Independent samples *t* test	Lesbian women had higher SAS than heterosexual women	*d* = 0.46*
*Comparisons of self*-*acceptance of sexuality*						
Ifrah et al. (2018)	Lesbian women	Gay men	SAS: GIQ-AHS	Independent samples *t* test	Lesbian women had lower SAS than gay men	*d* = 0.50*
Rosario et al. (2006)	Gay/lesbian women and men	Bisexual women and men	SAS: one items developed by the authors	ANOVA	At two time points, gay/lesbian individuals had significantly higher SAS than bisexual individual	
					6-month follow-up	*η*^2^ = .07**
					12-month follow-up	*η*^2^ = .04**
Rosario et al. (2009)	Lesbian women	Bisexual women	SAS: one items developed by the authors	ANOVA	Time 1: (6-month follow-up)	
					No significant differences in SAS between masculine lesbian women, feminine lesbian women, or feminine bisexual women	*η*^2^ = .08
					Time 2: (12-month follow-up)	
					Masculine lesbian women had higher SAS than feminine bisexual women	*η*^2^ = .17*
					There was no significant difference between these groups and feminine lesbian women	NSA
Shilo and Savaya (2011)	Gay/lesbian women and men	Bisexual women and men	SAS: SAQ	Independent samples *t* test	Bisexual women and men had significantly lower SAS than lesbian/gay women and men	*d* = 7.17**

Statistically significant results are bolded. *p < .05; **p < .01. NSA = non-significant association, effect size not specified. Effect sizes: *d *= Cohen’s *d. η*^2^ = eta squared. SAS = Self-Acceptance of Sexuality. GSA = General Self-Acceptance. DSA = Dignan (1965) Self-Acceptance Scale; GIQ-AHS = Gay Identity Questionnaire, Acceptance of Homosexuality Subscale (Brady & Busse, 1994); PWS-SA = Psychological Well-being Scale, Self-Acceptance Subscale (Ryff, 1989); SAQ = Self-Acceptance Questionnaire (Elizur & Mintzer, 2001)

## Summary of Study Findings and Discussion

### Self-Acceptance and Distal Stressors

Most of the relevant included studies found that poorer LGBQ+ self-acceptance was associated with a lack of acceptance of one’s sexuality by family and friends. Two studies conducted in the U.S. and one in Israel found that self-acceptance of sexuality had a small negative relationship with lower levels of acceptance of participants’ LGBQ+ sexuality by either their family (*r* = − .19 to − .24) or friends (*r* = − .23 to − .25; Hershberger & D’Augelli, 1995; Leserman, Disantostefano, Perkins, & Evans, 1994; Shilo & Savaya, 2011). Elizur and Mintzer (2001, 2003) further found that the negative relationship between a lack of friends’ acceptance and LGBQ+ self-acceptance upheld while controlling for a lack of family acceptance (*r* = − .22). Overall, these findings are supported by theoretical approaches, which would suggest that a lack of support from others has a deleterious effect on self-acceptance for individuals who identify as LGBQ+ (Cass, 1979; Elizur & Mintzer, 2001; Meyer, 2003).

In contrast, Elizur and Mintzer (2001, 2003) did not find a significant association between family acceptance and self-acceptance of one’s sexuality (*r* = − .08) within their study conducted in Israel, unlike other studies conducted in Israel (*r* = − .24; Shilo & Savaya, 2011) or the U.S. (*r* = − .19; Leserman et al., 1994). While both studies conducted in Israel utilized similar measures and had similar methodological strengths, different findings may have been a consequence of the data being collected from varied samples. For example, Shilo and Savaya (2011) included participants who identified as lesbian, gay, or bisexual and were male or female with an average age of 18 years. Conversely, Elizur and Mintzer (2001, 2003) only included participants identifying as gay and male, with an average age of 32 years. It may be, as one example, that family acceptance of one’s minority sexuality is more important for younger participants who are still undergoing identity development. Future studies could investigate whether family acceptance of sexuality is more strongly associated with self-acceptance for younger than older LGBQ+ people.

Only one study included in this review investigated the relationship between self-acceptance of sexuality and victimization. Hershberger and D’Augelli’s (1995) U.S. study found that self-acceptance of sexuality had small significant positive relationships with aspects of self-reported heterosexist victimization, including the frequency of experienced verbal insults and threats (*r* = .17), and having personal property damaged, being chased, followed, or spat on (*r* = .21). No significant relationship was found between self-acceptance of sexuality and the frequency of experienced physical or sexual assault related to one’s sexuality (*r* = .14; Hershberger & D’Augelli, 1995). This is contrary to theoretical approaches, which would consider heterosexist victimization to have a deleterious impact on self-acceptance of sexuality (Cass, 1979; Elizur & Mintzer, 2001; Meyer, 2003). However, it is possible that there is a bidirectional relationship; for example, that people who are more self-accepting may be less likely to conceal their sexuality and therefore be exposed to greater risk of victimization. This finding may have also been impacted by limitations in the sampling procedure. For example, the sample was recruited from LGBTQ+ youth community centers. Individuals attending these centers are likely to be more self-accepting and have access to supportive LGBTQ+ peers, which may mean they had increased opportunities to process and reappraise the impact of their experiences of victimization on self-acceptance. Furthermore, while many of the samples reported experiencing at least one incident of verbal victimization, few reported experiencing physical victimization, which may not have provided a sufficient range of experience to reveal an association. Finally, victimization was measured using one item for a combination of different types of victimization (e.g., personal property damaged and being chased, followed, or spat on, all within one item), which may have confused participants if they experienced only one part of this item and not others. Therefore, further research is needed to clarify the relationship between LGBQ+ self-acceptance and victimization.

### Self-Acceptance and Proximal Stressors

Sexuality self-acceptance had small to large negative associations with lower disclosure of one’s sexuality to others in general (*r* = − .27 to − .52) and to family members (*r* = – .25 to – .44) in four studies conducted in Israel and two in the U.S. (Elizur & Mintzer, 2001, 2003; Hershberger & D’Augelli, 1995; Ifrah, Shenkman, & Shmotkin, 2018; Leserman et al., 1994; Shilo & Savaya, 2011). Internalized heterosexism was also found to have a significant negative relationship with self-acceptance of sexuality in one study conducted in Russia (*ρ* = − .50; Yanykin & Nasledov, 2017). It has been suggested that internalized heterosexism often contributes to poor self-acceptance, and this in turn is associated with increased concealment of sexuality (e.g., Elizur & Mintzer, 2001; Meyer, 2003). Furthermore, it is suggested that attempting disclosure within a heterosexist environment can have a negative impact on self-acceptance, whereas disclosure to supportive and accepting others can facilitate access to affirmative and supportive experiences, thus improving self-acceptance (Elizur & Mintzer, 2001; Pepping et al., 2018; Pitoňák, 2017). This would be consistent with the findings of one study that the relationship between disclosure to others in general and self-acceptance of sexuality was no longer significant when controlling for family and friends’ support (Elizur & Mintzer, 2003).

While the relationship between disclosure to others and LGBQ+ self-acceptance was investigated in a number of cross-sectional studies with varying degrees of methodological strengths, internalized heterosexism was only investigated in one study that has comparatively increased limitations in the quality of reporting and attempts to reduce bias in the sample. Therefore, the possible bidirectional relationship between LGBQ+ self-acceptance and these processes requires further research, including with longitudinal or intervention/experimental research methods.

### Self-Acceptance and Mental Health

The findings of this review generally suggest that self-acceptance of sexuality is associated with better mental health. Three studies conducted in Israel and one in the U.S. found that various measures of general psychological distress had a small to medium negative relationship with self-acceptance of sexuality (*r* = − .26 to − .46; Hershberger & D’Augelli, 1995; Shilo et al., 2015; Shilo & Mor, 2014; Shilo & Savaya, 2011). However, this was not the case in a study which measured distress using the Symptom Checklist-90-Revised (SCL-90-R) in a sample from Russia (*ρ* = − .10; Yanykin & Nasledov, 2017). Similarly, this study found no significant relationship between self-acceptance of sexuality and psychological well-being (*ρ* = .07), unlike three studies in Israel reporting a significant small positive relationship with self-acceptance of sexuality (*r* = .18 to –.23; Shilo et al., 2015; Shilo & Mor, 2014; Shilo & Savaya, 2011). Two Israeli studies found that the relationship between self-acceptance of sexuality, psychological distress, and well-being was maintained while controlling for demographic variables, outness about sexuality, distal and proximal harassment, family and friends’ support, LGBQ+ connectedness, religiosity, victimization, and being in a steady relationship (Shilo et al., 2015; Shilo & Mor, 2014).

Only one study, conducted in the U.S., investigated the association between depression and self-acceptance of sexuality, and provided mixed evidence regarding this relationship (Leserman et al., 1994). For HIV-negative gay men, depression had a negative relationship with self-acceptance of sexuality, and this relationship was maintained while controlling for age, race, and education (Leserman et al., 1994). However, for HIV-positive gay men, the same study found that there was no significant relationship between depression and self-acceptance. It is possible that for HIV-positive gay men, factors associated with their HIV-positive status may have a larger impact on potential depression symptoms than self-acceptance. Furthermore, such relationships may have been very different at the time of the study compared to the current situation, so this requires further research.

Only one study investigated self-acceptance of sexuality in relation to suicidality and found no significant relationship with suicidal ideation (*r* = − .05) or attempts (*r *= .10; Hershberger & D’Augelli, 1995). It is possible that suicidality may be less strongly associated with self-acceptance than with stressful events, such as LGBQ+ victimization (Gnan et al., 2019; Hershberger & D’Augelli, 1995). It is also possible that people whose self-acceptance is so problematic that they consider suicide will be less likely to report identifying with a minority sexual orientation in research, and those who completed suicide cannot be included in many research designs. However, Herberger and D’Augelli’s (1995) study had limitations regarding the reporting of their methods and findings, and they did not use validated measures of suicidality or self-acceptance; thus, further research is needed with improved measurement instruments and methodological quality.

Overall, the findings that self-acceptance of sexuality was associated with lower psychological distress and greater well-being, as well as lower depression symptoms in HIV-negative gay men, are consistent with suggestions from past research that poor self-acceptance of one’s minority sexuality may negatively affect mental health (Meyer, 2003; Vincke & Bolton, 1994) and that greater self-acceptance may reduce mental health difficulties by buffering the negative impact of heterosexism (Aristegui et al., 2018; Elizur & Mintzer, 2001; Hershberger & D’Augelli, 1995). However, the included studies investigated only a limited number of mental health outcomes and findings were not replicated with all self-report measures of psychological distress, well-being, or depression symptoms employed. For example, no relationship was found between self-acceptance and the SCL-90-R in a Russian sample (Yanykin & Nasledov, 2017) or the Profile of Mood States, Depression subscale in a U.S. sample (Leserman et al., 1994). However, it is not possible to draw any firm conclusions regarding these variations in findings as they may reflect sample differences; for example, no relationship between self-acceptance and psychological distress in a Russian sample may be a result of sampling bias. For instance, the participants willing to access and take part in research regarding their LGBQ+ sexual identity—in a culture with high levels of anti-LGBQ+ narratives and policies—may only do so if they had very high levels of self-acceptance and well-being. This is supported by more than half of participants within Yanykin and Nasledov’s (2017) study having the highest possible scores for self-acceptance and lowest possible for psychological distress. However, further research is needed to explore this suggestion.

### Self-Acceptance Comparisons between Different Sexual Orientations

One study found that lesbian women had significantly lower self-acceptance of sexuality than gay men in a young adult sample from Israel, with a medium effect (*d* = 0.50; Ifrah et al., 2018). Another study conducted with adolescents and young adults from New York found that those who identified as gay or lesbian had significantly higher self-acceptance of sexuality than those who identified as bisexual over two time points with, small to medium effects (6 months: *η*^2^ = .07 and 12 months: *η*^2^ = .04; Rosario, Schrimshaw, Hunter, & Braun, 2006). In a subsample of female participants from Rosario et al. (2006), lesbian women who identified as masculine had significantly higher self-acceptance of sexuality than bisexual women who identified as feminine at a 12-month follow-up (large effect; *η*^2^ = .17), but there was no significant difference between these groups at the 6-month follow-up (*η*^2^ = .08; Rosario, Schrimshaw, Hunter, & Levy-Warren, 2009). There was also no significant difference between these groups and a sample of lesbian women who identified as feminine at both time points (Rosario et al., 2009). Finally, a study conducted in Israel with adolescents and young adults who identified as male or female found that bisexual individuals had lower self-acceptance of sexuality than lesbian or gay participants, with a large effect (*d* = 7.17; Shilo & Savaya, 2011). These findings support theory and research, which suggests that bisexual individuals and lesbian women are at increased risk of minority stressors related to their sexual orientation and other aspects of identity (e.g., gender conformity) compared with gay men, and thus, they may experience more difficulties with identity development (Feinstein & Dyar, 2017; Hequembourg & Brallier, 2009; Meyer, 2003). However, further studies are needed to confirm these differences using more robust sampling procedures and consistent self-acceptance measures.

For studies comparing LGBQ+ individuals with heterosexual participants, the findings of one study conducted in Israel and another in the U.S., with good methodological quality, demonstrated that non-heterosexual participants had lower levels of general self-acceptance compared with heterosexual individuals, with small to medium effects (*d* = 0.20–0.44; Gil, 2007; Riggle, Rostosky, & Danner, 2009). Conversely, another study conducted in the UK, with lower methodological quality, found that female participants identifying as LGBQ+ had *higher* general self-acceptance than female heterosexual participants, with a medium effect size (*d* = 0.46; Siegelman, 1979). It is difficult to interpret these contrasting findings due to the differences in study quality and self-report measures utilized. It is possible, however, that the LGBQ+ participants willing to take part in such research in the different social climate of the 1970s may have been those who tended to have higher self-acceptance. Overall, however, the findings that LGBQ+ individuals have lower general self-acceptance compared to heterosexual groups are congruent with theory, which suggests that the exposure to minority stressors leaves LGBQ+ individuals more vulnerable to difficulties with mental health and well-being—including difficulties with processes such as self-acceptance—compared to their heterosexual peers (Meyer, 2003; Pitoňák, 2017; Riggle et al., 2009).

### Included Study Characteristics and Methodological Quality

The methodological quality of the included studies and attempts to reduce bias varied (Appendix 2 for AXIS tool ratings), which precluded firm conclusions about cross-study comparisons. An important general limitation was that all included studies were cross-sectional, and most were correlational. This prevents conclusions regarding any possible causal direction between the constructs investigated; for example, lower self-acceptance may result in greater perception of stigma processes rather than vice versa. Additionally, most studies utilized various self-report measures of self-acceptance and minority stressors. The variability in instruments impairs comparison of findings across the studies. It is also important to note that a third unmeasured variable, such as low mood, could influence self-reports of self-acceptance and minority stressors and thus explain the apparent associations found within this review (Althubaiti, 2016; Lewis, Cogburn, & Williams, 2015). Furthermore, the included studies investigated a limited number of minority stressors and mental health outcomes.

There was also an overreliance on using instruments that have seemingly not undergone the appropriate psychometric validation to ensure sufficient validity and reliability. Some of the included measures of sexuality self-acceptance (i.e., the Self-Acceptance Questionnaire; the Gay Identity Questionnaire, Acceptance of Homosexuality subscale; the modified Coping and Change Measure, and the modified Mayfield Internalized Homonegativity Inventory, Self-Acceptance subscale) had early evidence of factor structure and internal consistency, however, no known explicit investigation of test–retest stability, face/content validity, or construct validity. The singular items used to measure self-acceptance by the remaining studies (i.e., Hershberger & D’Augelli, 1995; Riggle et al., 2009) had no psychometric evaluation. Thus, the use of these measures may limit the reliability and validity of the findings. Many studies were excluded on the basis of using instruments to measure self-acceptance of sexuality that were originally designed to measure other distinct LGBQ-specific processes, such as internalized heterosexism, as their inclusion would impact the validity of the findings and cause conceptual confusion.

Most studies utilized convenience methods of sampling which are not likely to be representative of the target population and are more susceptible to bias. This is pertinent as individuals who chose not to take part may represent important subsamples of participants including those who are potentially difficult to engage in research regarding minority sexuality, such as those who are less self-accepting. However, it is acknowledged that it can be difficult to collect data on participants who decline to take part in research, to determine if they indeed represent a different subsample compared to those who participate. The majority of studies were also conducted in the U.S. or Israel, and most participants were White. This may hinder the ability to generalize findings outside of the countries or races/ethnicities examined within the included studies. Overall, studies included slightly more males than females, and there was no clear representation or attention to minority gender identities. Many studies also exclusively included lesbian or gay participants, which means there is a much smaller representation of bisexual and other minority sexual orientations. However, it is acknowledged that there are significant difficulties in recruiting marginalized populations such as LGBQ+ individuals, particularly those who are concealing their identity or are in the early stages of identity development, and where population-based research is not currently collecting sufficient demographic information for these groups. Therefore, considering all the methodological limitations, the conclusions drawn within this review should be treated as tentative and not generalized to under-studied sub-populations of the sexual and gender minority community.

### Strengths and Limitations of the Current Review

With regard to the limitations of the current review, firstly, the inclusion of studies only published as journal articles means there is likely an over-representation of studies finding significant associations between study variables, as non-significant findings are less likely to be published (Rothstein, Sutton, & Brorenstein, 2005). Additionally, the exclusion of qualitative studies may mean potentially informative findings were not included in this review. Secondly, the initial screening of titles and abstracts was completed by only one researcher, which may mean potentially relevant articles may have been missed. Thirdly, there was a large amount of heterogeneity between different study populations and designs. For example, year of publication ranged from the 1970s until the 2010s, which represent very different social climates for LGBQ+ individuals and the self-acceptance of their minority identity. Measures of self-acceptance, sexual orientation, minority stressors, and mental health were not consistent across studies, which limited comparisons of findings.

Finally, many potentially relevant studies were excluded due to using measures originally designed to measure other variables such as internalized heterosexism, self-esteem, and other LGBQ-related processes, which the authors either adapted or used as a proxy for self-acceptance of sexuality. Studies were only included in this review if their self-acceptance of sexuality measures enquired directly about acceptance and/or comfort with one’s sexuality. However, the search strategy would not necessarily have identified studies that assessed constructs closely related to self-acceptance, such as comfort with sexuality—unless the author explicitly stated that they were using this as a proxy measure for self-acceptance. This may mean that potentially informative studies may have been excluded. However, it should be noted that the use of measures not designed to measure self-acceptance results in conceptual confusion and potentially invalid conclusions. For example, theoretical distinctions have been made between internalized heterosexism and lack of self-acceptance, and therefore, the authors of this review suggest that future research into self-acceptance should only utilize measures specifically designed to measure this construct. Similarly, self-acceptance of sexuality shares some conceptual overlap with other constructs such as self-affirmation. Despite these potential similarities, self-affirmation likely includes positive feelings toward the self, whereas self-acceptance can represent a more neutral acceptance of one’s sexuality. Therefore, research investigating self-affirmation was not included in this review as self-affirmation measures rely on items enquiring about pride and liking toward one’s sexuality (e.g., Mohr & Kendra, 2011).

Despite these limitations, the operationalized and systematic search strategy, which is replicable; the double ratings of the full text reviews; the inclusion of methodological quality ratings that were rated by two independent researchers; and the large number of participants considered across the included studies constitute strengths of this review.

### Implications and Recommendations for Theory and Research

The findings of this review are mostly consistent with minority stress and identity development theories, which suggest that minority stressors negatively impact self-acceptance and that self-acceptance may ameliorate the negative effect of minority stress (Cass, 1979; Elizur & Mintzer, 2001; Meyer, 2003). However, minority stress theory specifies little about the possible mechanisms as to how this occurs. Identity development theories elaborate further about how self-acceptance processes are part of building a positive identity as a sexual minority; however, self-acceptance within these models is typically conflated with other identity development processes (e.g., disclosure) or minority stressors (e.g., internalized heterosexism). This limited theoretical attention to LGBQ+ self-acceptance processes may have contributed to the under-representation of this construct in research and the use of heterogeneous measurement instruments. Theories focusing on or including LGBQ+ self-acceptance may benefit from drawing on theories of general self-acceptance processes which further detail proposed mechanisms of how self-acceptance interacts with well-being, for example via a non-judgmental and willing approach to managing distressing emotional and relational experiences (e.g., Bernard, 2013; Ryff, 2014; Williams & Lynn, 2010). Resilience theories also suggest that self-acceptance has the potential to be an important process for managing minority stressors (e.g., Aristegui et al., 2018; Mimiaga et al., 2015), but this has not been adequately empirically tested.

Future research should utilize validated measures and longitudinal data to gain stronger information about possible causal relationships, alongside studies investigating mediating and moderating effects of self-acceptance. Additionally, future research should investigate a diverse range of minority stressors and mental health outcomes and assess which have the largest impact on self-acceptance, as well as any impact in the opposite direction. As sample differences may have contributed to discrepant findings within this review, future studies could also investigate differences in these associations between different cultures, age groups, sexual identities, or other sociodemographic groups. Samples in future research should additionally better investigate self-acceptance of sexuality in groups with sexual identities (e.g., pansexual) that are under-represented in the current research, to improve generalizability and reveal any differences between these groups. Sexuality and gender self-acceptance should also be specifically investigated in LGBQ+ individuals who also have a minority gender identity. Qualitative research may also provide useful information about possible psychological processes involved in sexuality self-acceptance, which requires further understanding if people can be adequately supported when they experience self-acceptance difficulties. Future systematic reviews exploring self-acceptance of sexuality may consider also including search terms for closely related processes, such as comfort with sexuality, and including qualitative studies to be more inclusive.

### Implications and Recommendations for Clinical Practice

The relationship between self-acceptance, minority stressors, and mental health difficulties may suggest that low levels of self-acceptance should be addressed in interventions with LGBQ+ individuals aiming to improve psychological adjustment and well-being (American Psychological Association, 2012). Indeed, it has been suggested that self-acceptance is addressed in psychological interventions developed specifically for those identifying as LGBQ+, but there is no known evidence that these improve self-acceptance (e.g., Mustanski, Greene, Ryan, & Whitton, 2015; Pachankis, Hatzenbuehler, Rendina, Safren, & Parsons, 2015; Safren et al., 2014). The American Psychological Association (2012) guidelines for psychological interventions with LGBQ+ individuals suggest self-acceptance can be improved by providing a supportive and bias-free environment to discuss relevant issues. More specifically, Mustanski et al. (2015) suggest that clinicians may help to promote LGBQ+ self-acceptance by supporting the person to build an understanding of their sexuality, helping them to explore how this fits within their wider social context, challenging internalized heterosexism, supporting them to connect with others with similar experiences, supporting problem solving around disclosing to others, and coping with experiences of heterosexism. Other LGBQ-affirmative interventions suggest that cognitive behavioral strategies can be used to build awareness of the negative effects of minority stress on one’s mental health and address unhelpful beliefs and behaviors, to help improve well-being in LGBQ+ populations (Lin, Israel, & Ryan, 2019; Pachankis, 2014).

### Conclusions

In summary, the findings of this review tentatively suggest that self-acceptance of sexuality is negatively associated with the presence of some distal stressors (e.g., lack of acceptance by family and friends), proximal stressors (e.g., lack of disclosure to others), and mental health difficulties (e.g., greater global distress and depression, and lower psychological well-being). In contrast to theory, the findings of this review suggest self-acceptance of sexuality may be positively associated with heterosexist victimization and has no association with suicidality, although these results each represent the findings from only one study with low methodological quality. Additionally, on average individuals identifying as LGBQ+ had lower general self-acceptance than heterosexual people. Moreover, individuals identifying as bisexual also had lower self-acceptance of sexuality than lesbian and gay participants, and lesbian women had lower self-acceptance than gay men. These findings are largely consistent with minority stress and identity development theories. Unfortunately, the methodological limitations of the included studies, including their cross-sectional designs, limit the ability to draw firm conclusions. The findings suggest there is a clear need for further and more robust research investigating both self-acceptance of sexuality and general self-acceptance within LGBQ+ populations.

## Appendix 1: Search Terms

Search terms and Boolean operators developed for OvidSP are presented in Appendix 1. Boolean operators were changed to their equivalent for Web of Science.

See Table 4.

**Table 4:** Search terms

*“accept*” ADJ12 (“LGB*” OR gay or lesbian OR “bisex*” OR queer OR “pansex*” OR “homosex*” OR “sexual minority” OR “same sex attraction” OR “sexual orientation” OR sexuality OR “men who have sex with men” OR “women who have sex with women” OR non*-*heterosexual OR “sexual preference” OR “sexual identity”)*	
*OR*	
*“self*-*accept*” and (“LGB*” OR gay OR lesbian OR “bisex*” OR queer OR “pansex*” OR “homosex*” OR “sexual minority” OR “same sex attraction” OR “sexual orientation” OR sexuality OR “men who have sex with men” OR “women who have sex with women” OR non*-*heterosexual OR “sexual preference” OR “sexual identity”)*	

ADJ12 = adjacent within 12 words

## Appendix 2: Methodological Quality Ratings

See Table 5.

**Table 5:** Quality ratings of the included studies’ methodology using the AXIS Critical Appraisal Tool

References	Criteria																				Scores			
	1	2	3	4	5	6	7	8	9	10	11	12	13	14	15	16	17	18	19	20	Report	Design	Bias	Total
Elizur and Mintzer (2001, 2003)	+	+	−	+	−	−	−	+	+	+	+	+	−	−	+	+	+	+	−	+	7	4	2	13
Gil (2007)	+	+	−	+	−	−	+	+	+	+	+	+	+	−	+	+	+	+	?	+	7	4	4	15
Hershberger and D’Augelli (1995)	+	+	−	+	−	−	−	+	−	−	−	−	−	−	+	+	+	+	?	+	4	4	1	9
Ifrah et al. (2018)	+	+	−	+	−	−	−	+	+	+	+	+	+	−	+	+	+	+	?	+	7	4	3	13
Leserman et al. (1994)	+	+	−	+	−	−	−	+	−	+	+	+	?	−	+	+	+	−	?	+	6	4	1	11
Pepping et al. (2018)	+	+	−	+	−	−	+	−	+	+	−	+	+	+	+	+	+	+	?	+	6	3	5	14
Riggle et al. (2009)	+	+	−	+	+	+	−	+	+	+	+	+	?	−	+	+	+	+	?	?	7	4	3	14
Rosario et al. (2006, 2009)	+	+	−	+	−	−	−	+	+	+	+	−	?	?	?	+	+	+	+	+	5	2	1	8
Shilo and Mor (2014)	+	+	−	+	−	−	−	+	+	+	+	−	?	−	+	+	+	+	?	+	6	4	2	12
Shilo and Savaya (2011)	+	+	−	+	−	−	−	+	+	+	+	+	?	−	+	+	+	+	?	+	7	4	2	13
Shilo et al. (2015)	+	+	−	+	−	−	−	+	+	+	+	−	?	−	+	+	+	+	?	+	6	4	2	12
Yanykin and Nasledov (2017)	+	+	+	+	−	−	−	+	+	+	−	−	?	−	+	+	+	−	?	?	4	4	2	10
Total +	12	12	1	12	1	1	2	11	10	11	9	7	3	1	11	12	12	10	1	10				

+ = study adequately meets this criteria; − = study does not adequately meet criteria; ? = unable to determine whether study meets criteria. Items 13 and 19 are reverse scored. Report = Quality of Reporting Score: items 1 (aims/objectives reported), 4 (target population defined), 10 (statistical methods reported), 11 (study methods reported), 12 (basic data reported), 16 (results presented for all analyses), 18 (limitations discussed); Design = Study Design Quality: items 2 (appropriate study design), 3 (sample size justified), 5 (sample representative of target population), 8 (measures are appropriate to the aims), 17 (discussions and conclusions justified), 19 (conflicts of interest), 20 (ethical approval/practice); Bias = attempts to reduce bias: items 6 (selection process for participants likely result in a representative sample), 7 (efforts to address/categorize non-responders), 9 (variables measured with validated measures), 13 (response biased raised concerns), 14 (information about non-responders described), 15 (results were internally consistent); Total = total score: all items
